# Screening for celiac disease in Danish adults

**DOI:** 10.3109/00365521.2015.1010571

**Published:** 2015-02-17

**Authors:** Anna Horwitz, Tea Skaaby, Line Lund Kårhus, Peter Schwarz, Torben Jørgensen, Jüri J. Rumessen, Allan Linneberg

**Affiliations:** ^a^ ^1^Research Centre for Prevention and Health, The Capital Region, University of Copenhagen, Copenhagen, Denmark; ^b^ ^2^Department of Neuroscience and Pharmacology, Center for Healthy Ageing, University of Copenhagen, Copenhagen, Denmark; ^c^ ^3^Research Centre for Ageing and Osteoporosis, Departments of Medicine M and Diagnostics, Glostrup Hospital, University of Copenhagen, Copenhagen, Denmark; ^d^ ^4^Faculty of Health and Medical Sciences, University of Copenhagen, Copenhagen, Denmark; ^e^ ^5^Faculty of Medicine, Aalborg University, Aalborg, Denmark; ^f^ ^6^HR-Research Unit and Department of Gastroenterology, Gentofte Hospital, University of Copenhagen, Copenhagen, Denmark; ^g^ ^7^Department of Clinical Experimental Research, Glostrup University Hospital, Glostrup, Denmark

**Keywords:** Celiac disease, epidemiology, prevalence, screening

## Abstract

***Objective.*** The prevalence of celiac disease (CD) as recorded in the Danish National Patient Registry is ∼50/100,000 persons. This is much lower than the reported prevalence of CD in other Nordic countries and underdiagnosis is suspected. Our aim was to estimate the prevalence of CD in a population-based study of Danish adults. ***Methods.*** A total of 2297 adults aged 24–76 years living in the southwestern part of Copenhagen were screened for CD by immunoglobulin (Ig)A and IgG antibodies to transglutaminases and deamidated gliadin. IgA/IgG-positive participants were invited to a clinical evaluation, including biopsies, by a gastroenterologist. ***Results.*** Of the invited 56 participants, 40 underwent a full clinical evaluation and 8 persons were diagnosed with CD; 2 of the 16 persons, who did not complete the clinical evaluation, were considered by experts to have probable CD. None of the above 56 participants had a known history of CD or a recorded diagnosis of CD in National Patient Registry. By combining cases of biopsy-proven CD (*n* = 8), probable CD (*n* = 2), and registry-recorded CD (*n* = 1), the prevalence of CD was estimated to be 479/100,000 (11/2297) persons (95% CI: 197–761). ***Conclusion.*** In this general adult population, the prevalence of CD as estimated by screening and clinical evaluation was 10 times higher than the registry-based prevalence of CD. Of 11 participants diagnosed with CD in our screening study, 10 were unaware of the diagnosis prior to the study. Thus, our study suggests that CD is markedly underdiagnosed in Danish adults.

## Introduction

Celiac disease (CD) is an autoimmune disease triggered by gluten in genetically susceptible individuals. CD is characterized by gastrointestinal symptoms, macroscopic and microscopic changes in the small bowel mucosa, malabsorption, and a wide range of extraintestinal manifestations [1]. The diagnosis of CD is based on determination of CD-specific biomarkers (antibodies), histological examination of duodenal biopsies, and improvement following initiation of gluten-free diet [2].

The reported prevalence of CD varies substantially and large differences have been observed even within short geographical distances [3]. Scandinavia is regarded as a high-prevalence area. In Sweden, CD affects ∼1000–3000/100,000 children [4, 5]; 530/100,000 adults; and 270/100,000 blood donors [6, 7]. One study from Norway screened healthy blood donors and found a prevalence of 290/100,000 persons [8]. A Finnish screening study in adults found that the prevalence was 1740 and 1240/100,000 persons as assessed by CD-specific antibodies and biopsy, respectively [9].

No previous study has screened for CD by using CD antibodies and clinical evaluation, including endoscopy, in a Danish general adult population. A Danish registry-based study including all Danish inhabitants (both children and adults) found that the recorded prevalence of diagnosed CD was 55/100,000 persons [10]. A similar nationwide registry-based study of all Danish children found that the prevalence of CD was 80/100,000 children [11]. A Danish registry-based study (based on hospital records) including all Danish adults in Copenhagen during the period between 1976 and 1991 reported a prevalence of 46/100,000 persons [12]. Several studies have indicated that CD is underdiagnosed. For example, a Swedish study showed that 8 out of 10 screen-detected CD patients were undiagnosed [6]. The extent of underdiagnosis in Denmark is not known.

The present study aims at investigating the prevalence of CD in a Danish adult population by CD-specific antibody screening and subsequent clinical examination including small intestinal biopsy in screen-test-positive individuals.

## Methods

### *Study population*

**Figure 1.**
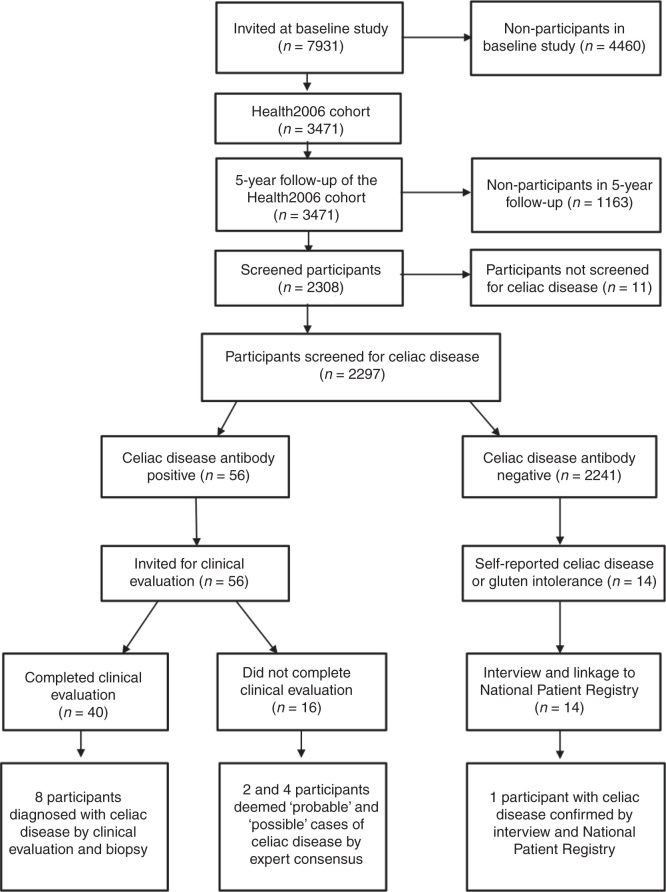
Diagram of study design and flow of participants in the Health2006 cohort.

The study was based on the 5-year follow-up of the Health2006 cohort. A detailed description of the baseline examination has been published elsewhere [13]. The participants invited to the baseline study were drawn as a random sample of 7931 persons from the background population aged 18–69 years, living in 11 municipalities in the southwestern part of Copenhagen. A total of 3471 persons (44.7%) participated in the baseline study between June 2006 and June 2008. In 2011–2012, all eligible participants in the Health2006 baseline study were invited to a 5-year follow-up examination including essentially the same study protocol [14] with the addition of screening for CD by measurements of CD biomarkers. A total of 3405 participants were eligible for invitation (21 had emigrated and 45 died) and 2308 (45.8% men) participants were reexamined between November 2011 and November 2012. The mean age at the follow-up examination was 55.7 years (range: 24–76 years). Baseline characteristics of participants and non-participants in the 5-year follow-up are shown in Table I. Informed written consent was obtained from all participants prior to participation. The Ethics Committee of the Capital Region of Denmark (code H-3-2011-081) approved the study. A diagram of the study design is shown in Figure 1.

**Table I.** **Baseline characteristics of participants in the 5-year follow up of the Health2006 cohort study.**

	Participants in follow up (*n* = 2308)	Non-participants in follow up (*n* = 1163)	*p*-Value
Gender	% (n/n total)	% (n/n total)	
Female	54.2 (1250/2308)	57.4 (668/1163)	*p* = 0.067 #
Male	45.8 (1058/2308)	42.6 (495/1163)	
Age at baseline	% (n/n total)	% (n/n total)	
15–34	12.7 (294/2308)	20.4 (237/1163)	*p* < 0.001 #
35–54	47.8 (1104/2308)	43.8 (509/1163)	
55+	39.4 (910/2308)	35.9 (417/1163)	
Employment status	% (n/n total)	% (n/n total)	
Employed or self-employed	76.6 (1745/2279)	68.2 (775/1137)	*p* < 0.001 #
Have been employed	22.3 (509/2279)	29.1 (331/1137)	
Have never been employed	1.1 (25/2279)	2.7 (31/1137)	
Smoking status	% (n/n total)	% (n/n total)	
Current daily smoker	17.4 (399/2290)	32.6 (374/1147)	*p* < 0.001 #
Occasional smoker	3.6 (82/2290)	2.7 (31/1147)	
Past smoker	34.2 (784/2290)	29.0 (332/1147)	
Never-smoker	44.8 (1025/2290)	35.8 (410/1147)	
Anthropometry	Mean (95% CI)	Mean (95% CI)	
Waist circumference in cm	88.0 (87.5–88.5)	90.1 (89.2–90.9)	*p* <0.001§
BMI in kg/m^2^	25.7 (25.5–25.9)	26.4 (26.1–26.7)	*p* <0.001§
Alcohol consumption	Median (IQR)	Median (IQR)	
Units per week in past 12 months	7 (3–14)	6 (2–13)	*p* = 0.007 *
	% (n/n total)	% (n/n total)	
Non-drinkers, past 12 months	3.9 (88/2285)	8.2 (93/1141)	*p* <0.001 #

# Chi-square test; * Wilcoxon-Mann–Whitney test; § Independent samples *t*-test. Abbreviations: BMI = Body mass index; IQR = Interquartile range.

Participants completed a self-administered questionnaire on, for example, health, diagnoses and symptoms of diseases, lifestyles, and socioeconomic factors. The questionnaire included the following question: ‘Has a doctor ever told you that you have or have had CD (gluten intolerance)?’ All 7931 members of the Health2006 cohort were linked using the unique personal identification number to the Danish National Patient Registry that holds information on diagnoses from all hospital admissions since 1977 and followed from the start of the register to 31 December 2011. Since 1995, diagnoses for outpatients are also included [15]. Diagnoses included in the register from 1977 to 1993 were classified according to the Danish version of the Eighth revision of the International Classification of Diseases (ICD-8). Since 1994, the ICD-10 version has been used. A registry-based diagnosis of CD was defined by the ICD-10 code K90.0 (before 1994: ICD-8 code 269.0).

### *Measurements of CD antibodies*

All participants in the 5-year follow up of the Health2006 cohort were screened for CD antibodies by using the Elia™ Celikey® tissue transglutaminase (TTG) anti-immunoglobulin(Ig)A assay and deamidated gliadin peptide (DGP) anti-IgA and anti-IgG assays [16, 17]. In participants with undetectable levels of IgA against TTG, suggestive of selective IgA deficiency, which is known to be associated with high risk of CD, IgG class antibodies against TTG were determined. The coefficient of variation (CV) of these assays as reported by the laboratory that performed the analyses was 7–8%. The Celikey assay system has proven its diagnostic efficacy in several studies. In particular, determination of anti-IgA against TTG, the major autoantigen in CD, has shown a high sensitivity and specificity in the diagnosis of CD [18, 19, 20]. The following cut-off values were used to define positivity and risk of CD: IgA-DGP ≥10.0 U/ml; IgG-DGP ≥10.0 U/ml; IgA-TTG ≥7.0 U/ml; and IgG-TTG ≥7.0 U/ml [16]. Human lymphocyte antigen (HLA) class variants known to be strongly associated with CD (HLA-DQ2 and HLA-DQ8) were determined [21]. All measurements of CD biomarkers were performed at Thermo Fisher Scientific, ImmunoDiagnostics (formerly Phadia), Allerød, Denmark.

### *Clinical examination of screen-test-positive participants*

All subjects who were positive to at least one of the serological CD biomarkers were contacted by a physician (T Skaaby) and invited to a clinical examination by a gastroenterologist (J Rumessen) at the Department of Gastroenterology, Gentofte Hospital, Denmark. The examination program was performed according to the standard program for evaluation of patients admitted to the department with suspicion of CD. This included additional blood tests, glucose and lactose breath tests, and gastroscopy, including four biopsies from the proximal duodenal mucosa. The biopsies were assessed by pathologists and classified according to the Marsh–Oberhuber classification [22].

Bone mineral density (BMD) was measured as part of the examination program. BMD-T-score was obtained at the lumbar spine (L1–L4) and total hip using a Hologic Discovery DXA scanner (Hologic, Bedford, MA, USA) by the same trained technologist. The Z- and T-score is used in the operational definition of osteoporosis in World Health Organization. Osteoporosis is defined as BMD (in g/cm^2^) 2.5 standard deviation below the reference material, that is, aged-matched healthy young individuals. The CV for all BMD measurements varied from 0.5% to 3% (Hologic, Bedford, MA, USA). Osteopenia and osteoporosis were defined as a T-score <−1 and −2.5, respectively, at the lumbar spine, or right or left hip.

### *Statistical analysis*

The 95% confidence intervals of prevalence (proportion; p) estimates were based on the binomial distribution (standard error calculated as the square root of p*(p-1)/n).

## Results

**Figure 2.**
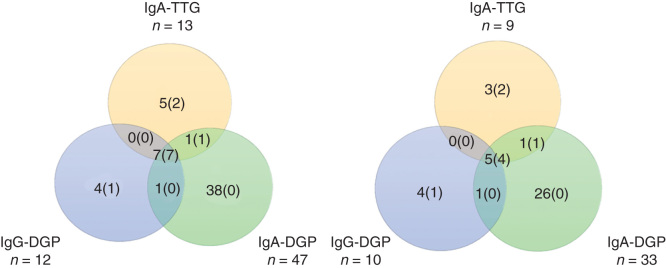
Venn diagram showing the relationship between positivity to IgA antibody to tissue transglutaminases, IgA to deamidated gliadin peptide, and IgG to deamidated gliadin peptide. A. A total of 56 participants were positive to at least one of the three antibody tests as shown in the Venn diagram. The figures in brackets represent number of participants who were diagnosed with celiac disease by clinical examination and biopsy and/or expert consensus. B. 40 antibody-positive participants who completed the clinical evaluation and intestinal biopsies. The figures in brackets represent numbers of participants diagnosed with celiac disease by clinical examination and positive histology. Abbreviation: Ig = Immunoglobulin.

A total of 2297 of the 2308 participants were screened for CD antibodies and 56 (2.4%) were positive to at least one of the antibodies. A Venn diagram of the relationship between positivity to IgA-TTG, IgA-DGP, and IgG-DGP is shown in Figure 2A. A total of 14 screened participants (out of the 2297 subjects) had undetectable levels of serum IgA-TTG and were in addition screened for IgG-TTG. None of them were positive (all <7 U/ml) for IgG-TTG. Figure 2B shows a Venn diagram of the relationship between positivity to IgA-TTG, IgA-DGP, and IgG-DGP among those 40 participants who underwent clinical evaluation. Out of these, eight participants were diagnosed with CD. As shown in Figure 2B, all eight participants diagnosed with CD were either IgA-TTG- or IgG-DGP-positive; none were only IgA-DGP-positive.

All 7931 persons who had been invited to the baseline study were linked to the Danish National Patient Registry and were followed in regard to diagnosis of CD. Among the 2297 screened participants, only 1 participant had a diagnosis of CD in the National Patient Registry. That participant also reported a diagnosis of CD in the questionnaire, but was not positive to any of the CD antibodies. One participant among the non-participants in the 5-year follow up had a diagnosis of CD in the National Patient Registry. Two persons among the non-participants in the baseline study had a diagnosis of CD in the National Patient Registry. Thus, there were four known cases of CD in the National Patient Registry in the whole cohort of 7931 persons (cumulated prevalence: 50/100,000 persons [95% confidence interval: 10–100 per 100,000 persons]). Figure 1 shows a diagram of the study design and flow of participants.

Among the screened participants, 14 participants reported in the questionnaire that they have or have had a diagnosis of CD or gluten intolerance. All of these were serologically screen-test negative. A physician (A Horwitz) performed telephone interviews of all 14 participants about how and by whom the diagnosis had been made, possible diets and treatments, relatives with similar conditions, and type and frequency of symptoms. Among the 14 interviewed participants, 10 could not confirm a history of CD or gluten intolerance. A diagnosis of CD was deemed plausible in the remaining four participants. However, three out of these four participants were HLA-DQ2- and HLA-DQ8-negative, leaving only one participant with a plausible diagnosis of CD. This patient was the same as the abovementioned participant, who had an ICD-10 code of K90.0 in the National Patient Registry.

All 56 serologically screen-test-positive participants were informed about the test results and invited to clinical evaluation. Out of these, 15 did not participate in the clinical evaluation. One participant died before the clinical evaluation. The cause of death was tonsil cancer. Of the 56 participants, 40 underwent a full clinical evaluation with intestinal biopsies. Table II shows the clinical characteristics of these participants. Of these 40 participants, 8 were diagnosed with CD based on clinical evaluation and histopathology. The Marsh classifications of the eight participants who were diagnosed with CD were as follows: 3B (*n* = 3), 3A (*n* = 2), 3C (*n* = 2), and 1 (*n* = 1). They were all HLA-DQ2- and/or HLA-DQ8-positive. The participant with Marsh 1 histological classification had marked lymphocyte infiltration, a high titer of IgA anti-TTG (115 U/ml), and showed good clinical improvement and associated decrease in IgA-TTG in response to gluten-free diet. Participants diagnosed with CD generally had few, if any, gastrointestinal symptoms (Table III). None of the participants diagnosed with CD in the clinical evaluation in our study had a known history of CD or a recorded diagnosis of CD in the National Patient Registry.

**Table II.** **Characteristics of 56 celiac disease antibody-positive participants according to whether they were diagnosed with celiac disease or not.**

		Participants who completed clinical evaluation and biopsy	
	All antibody-positive participants	Celiac disease not confirmed	Diagnosed with celiac disease
Number	56	32	8
Mean age in years	55.8 (26–74)	56.3 (26–74)	53.3 (29–73)
Antibody positivity			
IgA-TTG	13 (23.2)	2 (6.3)	7 (87.5)
IgA-DGP	47 (83.9)	27 (84.4)	5 (62.5)
IgG-DGP	12 (21.4)	5 (15.6)	5 (62.5)
IgG-TTG	0 (0)	0 (0)	0 (0)
HLA type			
+ DQ2/ − DQ8	28 (50.0)	14 (43.8)	6 (75.0)
+ DQ2/ + DQ8	4 (7.1)	2 (6.3)	2 (25.0)
− DQ2/ + DQ8	8 (14.3)	5 (15.6)	0 (0)
− DQ2/ − DQ8	16 (28.6)	11 (34.4)	0 (0)
Marsh classification			
0	-	30 (93.8)	0 (0)
1	-	2 (6.3)	1 (12.5)
2	-	0 (0)	0 (0)
3A	-	0 (0)	2 (25.0)
3B	-	0 (0)	3 (37.5)
3C	-	0 (0)	2 (25.0)
Breath tests			
Lactose intolerance	-	1	0
Glucose intolerance	-	0	0
BMD	*n* = 33	*n* = 26	*n* = 7
Total lumbar spine			
*t*-score	-	−0.27 (1.82)	−0.16 (1.60)
BMD (g/cm^2^)	-	1.04 (0.20)	1.05 (0.18)
Total left hip			
*t*-score	-	−0.50 (1.13)	−0.21 (0.78)
BMD (g/cm^2^)	-	0.93 (0.17)	0.95 (0.10)
Total right hip			
*t*-score	-	−0.52 (1.12)	−0.16 (0.97)
BMD (g/cm^2^)	-	0.92 (0.16)	0.96 (0.13)
Osteoporosis			
Osteoporosis	-	3	0
Osteopenia	-	9	2

Abbreviations: BMD = Bone mineral density; HLA = Human lymphocyte antigen; IgA-TTG = IgA antibody to tissue transglutaminase; IgA-DGP = IgA to deamidated gliadin peptide; IgG-DGP = IgG to deamidated gliadin peptide.

**Table III.** **Symptoms and diseases among the participants, who completed the clinical examination.**

	Celiac disease not confirmed (*n* = 32) % (*n*/total n)	Diagnosed with celiac disease (*n* = 8) % (*n*/total *n*)
**Participants with clinical evaluation and biopsy (n= 40)**		
History of celiac disease in family	7.7 (2/26)	12.5 (1/8)
Failure to thrive in childhood	23.1 (6/26)	0.0 (0/8)
Tiredness	23.1 (6/26)	37.5 (3/8)
Weight loss	7.7 (2/26)	0.0 (0/8)
Diarrhea	11.5 (3/26)	0.0 (0/8)
Constipation	15.3 (4/26)	0.0 (0/8)
Alternating stools	11.5 (3/26)	12.5 (1/8)
Abdominal pain (at least weekly)	11.5 (3/26)	0 (0/8)
Rumbling in the stomach	26.9 (7/26)	12.5 (1/8)
Bloating	38.5 (10/26)	12.5 (1/8)
Flatulence	19.2 (5/26)	12.5 (1/8)
Nausea	0.0 (0/26)	0.0 (0/8)
Autoimmune diseases		
Myxedema	1	0
Dermatitis herpetiformis	0	0

An expert committee (J Rumessen, A Linneberg, and A Horwitz) scrutinized all 16 IgA/IgG screen-positive participants who did not participate in the clinical evaluation (Figure 1). Among these 16 participants, 2 and 4 persons were considered to have probable and possible CD, respectively, based on their IgA/IgG and HLA results.

By combining cases of biopsy-confirmed CD (*n* = 8), probable CD (*n* = 2), and registry-recorded CD (*n* = 1), the prevalence of CD was estimated to be 479/100,000 (11/2,297) persons (95% confidence interval: 197–761/100,000 persons).

BMD was measured on 33 of the screen-positive participants (Table II). Out of 26 participants in the non-CD group, 9 and 2 out of 7 in the group diagnosed with CD had osteopenia, only 3 in the non-CD group had osteoporosis.

## Discussion

In this population-based study of adults, we estimated the prevalence of CD to be ∼500/100,000 persons. Out of the 2297 screened participants, we diagnosed 10 new cases of CD without a known history of CD and identified one known case of CD.

A previous Danish register-based study found that the prevalence of CD was 55/100,000 persons, including both children and adults as assessed by linking the whole population of Denmark to the Danish National Patient Registry [10]. This figure is very similar to the prevalence of CD observed when linking the whole Health2006 cohort of 7931 adults with the National Patient Registry, which revealed four cases of CD, of which one was observed among the 2297 participants screened in the present study. Our registry-based estimate of recorded CD prevalence in the whole Health2006 cohort and in those screened for CD antibodies in the 5-year follow up of the cohort was 50/100,000 (4/7931) and 44/100,000 (1/2297) adults, respectively. The finding that these two figures were similar may indicate that the participants in the 5-year follow up were likely to be representative of the background population in the study area in regard to prevalence of CD. Furthermore, the fact that these estimates were relatively similar to the estimate obtained for the whole population of Denmark [10] may also support that our estimate of CD prevalence obtained by screening and clinical evaluation is not severely biased. The CD prevalence estimated by screening and clinical evaluation was 10 times higher than the registry-based prevalence of CD in Denmark. Accordingly, 10 out of 11 participants diagnosed with CD in our screening study were unaware of their disease prior to the study. Thus, our study suggests that CD is markedly underdiagnosed in Denmark. However, our study also indicated that these screen-detected cases of CD had mild or negligible symptoms. This might be a cause for the underdiagnosis, as many of the CD patients have silent CD. Other causes for underdiagnosis might be low awareness of CD or lack of consensus regarding serological tests in primary healthcare.

The prevalence of CD observed in our study could represent an underestimation, if persons with undiagnosed CD tend to avoid gluten-containing foods. It is plausible that persons who experience gastrointestinal symptoms following ingestion of gluten-containing foods tend to avoid such foods. Such behavior can cause false-negative CD antibody test results and negative histological results of biopsies from the intestinal mucosa.

The prevalence of CD observed in our study was within the range of prevalence observed in studies in other Scandinavian and Northern European countries. A Finnish screening study found a serologically verified prevalence of 1740/100,000 and a biopsy verified prevalence of 1240/100,000 [9]. In a multicenter study from Finland, Germany, Italy, and Northern Ireland, based on screening studies of healthy adult populations, using an anti-TTG-assay, the following CD-prevalence was reported: Finland (serologically verified: 1870/100,000; biopsy-verified: 730/100,000), Germany (serologically verified: 430/100,000; biopsy-verified: 120/100,000), Italy (serologically verified: 1360/100,000; biopsy-verified: 480/100,000), and Northern Ireland (serologically verified: 1590/100,000; biopsy-verified: 60/100,000) [23].

The costs of screening should be considered. A full serologic screening costs ∼ €45 per participant in Denmark (each CD antibody assay costs €15 per sample). The cost of a biopsy is (in diagnosis-related groups) ∼ €513. Thus, with the used screening algorithm, the cost of finding one patient with CD was ∼ €12,000. In this context, risk of complications and discomfort and anxiety related to the clinical examination should also be considered [24] and balanced against the benefit of an early diagnosis [24].

The occurrence of CD has been described by an iceberg metaphor. The classical phenotype of CD with abdominal pain, diarrhea, and malabsorption is more likely to be diagnosed, whereas the majority of patients remain undiagnosed “below the waterline” [25]. Participants diagnosed with CD in our study did not report frequent gastrointestinal symptoms, which may have contributed to the fact that they had remained undiagnosed prior to the study. The consequences of undiagnosed CD are not well described. Studies have shown that patients with CD have an increased risk of other autoimmune diseases and gastrointestinal cancer [26, 27, 28]. One study found a fourfold increased mortality in persons with untreated and undiagnosed CD [29], whereas others reported mortality rates to be relatively similar to those observed in the background population [2, 30, 31]. An early diagnosis of CD is important, since untreated CD may cause micronutrient deficiencies and complications such as osteoporosis, anemia, growth retardation, infertility, and neurological disorders [32, 33].

In conclusion, we found that the prevalence of CD as estimated by screening and clinical evaluation was 10 times higher than the registry-based prevalence of CD in Denmark. Of the 11 participants diagnosed with CD in our screening study, 10 were unaware of their disease prior to the study. Thus, our study suggests that CD is markedly underdiagnosed in Denmark.
